# A prospective study of two-dimensional ultrasonography combined with shear wave elastography for pregnancy-related diastasis recti abdominis

**DOI:** 10.3389/fphys.2024.1382982

**Published:** 2024-05-27

**Authors:** Li Wang, Ting Yun, Dong Zhang, Jianrong Zhong, Dan Yi, Wanxi Fu, Molin Li, Yunshan Zhang, Yuexiang Wang

**Affiliations:** ^1^ Graduate School of the PLA General Hospital, Beijing, China; ^2^ Department of Ultrasound, The Six Medical Center of Chinese PLA General Hospital, Beijing, China; ^3^ Orthopedics Department, The Six Medical Center of Chinese PLA General Hospital, Beijing, China; ^4^ Department of Gynaecology and Obstetrics, The Six Medical Center of Chinese PLA General Hospital, Beijing, China; ^5^ Department of Ultrasound, The First Medical Center of Chinese PLA General Hospital, Beijing, China

**Keywords:** ultrasonography, elasticity imaging techniques, rectus abdominis, inter-rectus distance, muscle thickness

## Abstract

**Objectives:**

To compare the inter-rectus distance (IRD), rectus abdominis thickness (RAT), and stiffness in women during pregnancy and postpartum and identify the risk and protective factors affecting diastasis recti abdominis (DRA).

**Materials and methods:**

A total of 171 pregnant women who volunteered to participate in this study were recruited. Using an ultrasonographic diagnostic instrument with shear wave elastography function, IRD, RAT and the Young’s modulus of the rectus abdominis muscles were measured at 12 weeks, 37 weeks of pregnancy, and 6 weeks postpartum.

**Results:**

The IRD at 37 weeks was significantly higher than that at 12 weeks and then decreased at 6 weeks postpartum, but it was still higher than that at 12 weeks (*p* < 0.001). RAT and Young’s modulus decreased significantly at 37 weeks compared with those at 12 weeks and then recovered at 6 weeks postpartum, but they were lower than those at 12 weeks (*p* < 0.001). IRD at 12 weeks was significantly higher in multiparae than in primiparae (*p* < 0.001). Moreover, positive correlation between the RAT and Young’s modulus of rectus abdominis muscles at 12 and 37 weeks of gestation and 6 weeks postpartum (*p* < 0.001) was observed. Multiple linear regression analysis showed that the regression equation was significant (f = 24.856, *p* < 001).

**Conclusion:**

Our study identified differences in IRD, thickness and stiffness of the rectus abdominis muscle between early and advanced pregnancy and the postpartum period. The risk and protective factors of DRA may guide pregnant women’s protection and treatment.

## Introduction

Diastasis recti abdominis (DRA) is an abnormal separation of the rectus abdominis muscles on both sides of the abdomen and manifests as an increase in the inter-rectus distance (IRD). This condition is common in the third trimester of pregnancy and after delivery (Fernandes da Mota et al., 2015). Controversy remains in the diagnostic criteria for DRA, and the currently recognized criterion (Bo et al., 2017) is that DRA can be diagnosed when the IRD is ≥ 2 fingers width or ≥2 cm. However, some researchers (Mota et al., 2018) have argued that this criterion should be relaxed to 2.8 cm. The incidence of DRA is almost 100% in the third trimester, which shrinks gradually after delivery and returns to the normal state in some women. However, if this is unresolved, it is known as postpartum DRA (Bursch, 1987; Boissonnault and Blaschak, 1988).

Thinning and weakness of the linea alba in patients with DRA is an important risk factor for abdominal wall hernia (Ha et al., 2016; Barbato et al., 2021). DRA can affect spinal and pelvic stability, alter overall body posture, and increase the risk of lumbago, chronic pelvic pain, and pelvic floor dysfunction (Benjamin et al., 2019; Eriksson Crommert et al., 2020). Some studies have reported that the body image and body satisfaction rate of patients with DRA are significantly lower than those of the general population (Keshwani et al., 2018; Gluppe et al., 2022).

The main methods currently used to diagnose DRA are palpation and caliper measurements, computed tomography (CT), magnetic resonance imaging (MRI), and ultrasonography. Owing to the simplicity and ease of palpation measurement, it is the commonly used method in clinical practice. However, it cannot accurately measure IRD (Sperstad et al., 2016; Keshwani et al., 2018). CT and MRI use powerful post-processing functions to accurately measure the extent, morphology, and degree of abdominal wall protrusion of DRA (Olsson et al., 2019). However, these techniques require sophisticated instruments and the investigation cost is high. These limitations severely limit their use (Emanuelsson et al., 2014). Ultrasonography offers the unique advantages of simplicity, no ionizing radiation, and high soft tissue resolution. Hence, ultrasonography is particularly suited to measuring IRD in pregnant women (Mendes Dde et al., 2007; Keshwani and McLean, 2015; Johnson et al., 2021).

Shear wave elastography (SWE) is an ultrasonographic technology that has emerged in recent years, which displays real-time images of tissue stiffness distribution and quantitatively analyzes the stiffness of human tissues (Athanasiou et al., 2010). SWE can indirectly reflect pathological changes caused by muscle hemorrhage and fibrosis by showing changes in stiffness after muscle injury. Pathological changes can also reflect muscle load, inflammation, and edema (Carolina et al., 2012). Moreover, Young’s modulus ( $E=3\rho c^{2}$ , where E represents the Young’s modulus, c represents shear wave speed, and ρ denotes the biological tissue density) of a muscle is positively correlated with muscle strength within a certain range (Soldos et al., 2021).

He et al. used SWE to measure rectus abdominis shear wave speed in 36 postpartum participants with DRA and 24 nulliparous healthy women. They found that rectus abdominis stiffness was significantly lower in the DRA group than in the healthy women group, with the difference being most pronounced at the umbilicus level. The rectus abdominis shear wave speed was positively correlated with IRD (r = 0.574). To conclude, He et al. reported that the application of SWE to the abdominal wall of patients with DRA is feasible (He et al., 2021).

To date, most ultrasonographic studies on DRA have used only conventional two-dimensional imaging. While the study conducted by He et al. also applied SWE to patients with DRA, this was a cross-sectional and not self-controlled study that compared nulliparous healthy women with women experiencing postpartum DRA. Therefore, the study could not eliminate the effect of pregnancy on rectus abdominis stiffness in both groups.

This study is a prospective study in which data were collected right from the first trimester of pregnancy and the women were followed until the postpartum period. Prenatal and postpartum examination sessions were used for self-control. The objective of our study was to compare IRD, rectus abdominis thickness (RAT), and stiffness in women during pregnancy and postpartum and to identify the risk and protective factors that affect DRA.

## Materials and methods

### Participants

The study was approved by the hospital ethics committee (approval number: HZKY-PJ-2021-31). Pregnant women in the first trimester were recruited from May 2021 to April 2022 to participate in this study. There were 171 pregnant women, and their age ranged 24–40 (31 ± 3) years. All women enrolled in the study were informed about the entire study content and the possible risks and benefits of participating in the study. They were enrolled on a voluntary basis and signed an informed consent form. The figures submitted in this article were approved by the participants. Inclusion criteria for the study were as follows: (1) women who were documented and delivered in our hospital; (2) women in their first trimester, aged 22–40 years, with a gestational age of 6–12 weeks. Exclusion criteria were (1) previous history of abdominal operation (including cesarean section); (2) multiple pregnancies or previous history of multiple pregnancies; (3) congenital dysplasia of the abdominal wall; (4) fetal abruption, premature labor, or transfer to another hospital and delivery during pregnancy; and (5) severe obesity, with a body mass index of ≥28.

### Instruments and methods

#### Instrumentation

A color Doppler ultrasonographic diagnostic instrument with a real-time SWE function was equipped with a linear array ultrasonographic probe with a bandwidth frequency of 5–14 MHz (the Resona 7 system, Mindray Imagine, Shen Zhen, China).

#### Methods

Ultrasonographic examination of the rectus abdominis muscle was performed at 12 and 37 weeks of gestation, and 6 weeks postpartum to measure IRD, rectus abdominis thickness (RAT), and Young’s modulus.

### Gray-scale ultrasonographic examination

**FIGURE 1:**
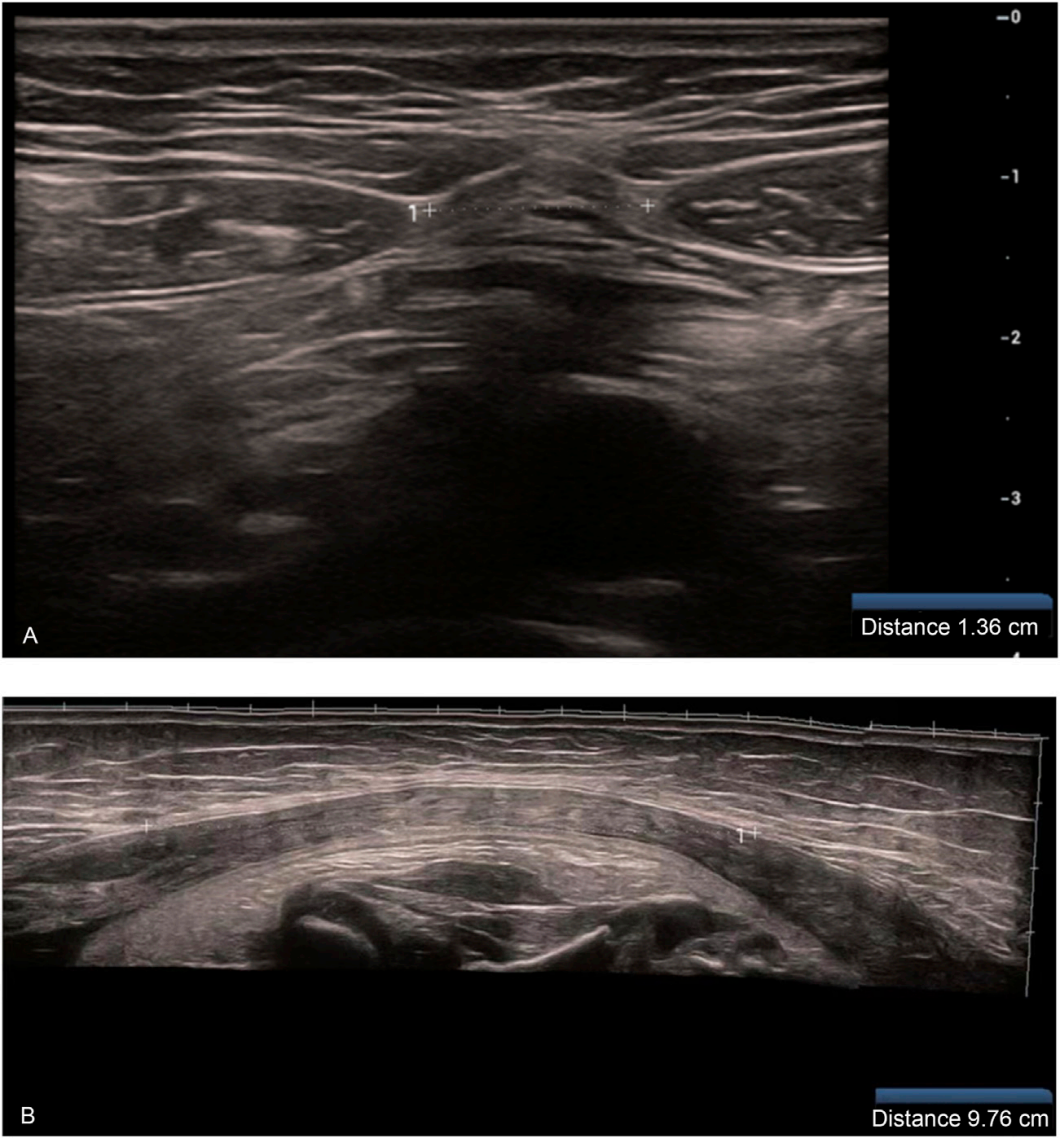
**(A)** Measurement of IRD at the upper umbilical border at the first trimester (12 weeks) **(B)** Measurement of IRD at the third trimester (37 weeks) (when the IRD exceeded the limit of what could be measured using a single cross-section of the probe, the wide-field imaging function was used for IRD measurement).

Gray-scale ultrasonographic measurement of IRD: The patient was placed in the supine position on the examination bed and in a naturally relaxed state. The examination area of the abdomen was fully exposed, and the bilateral rectus abdominis muscle was scanned in transverse and longitudinal sections using a linear array probe. The distance between the medial margins of the bilateral rectus abdominis muscle hyperechoic fascia was measured at the level of the superior umbilical border and recorded as IRD (Patricia et al., 2012; Nicole et al., 2019; Antonio et al., 2020). The mean value of three consecutive measurements was obtained (Figure 1).

**FIGURE 2:**
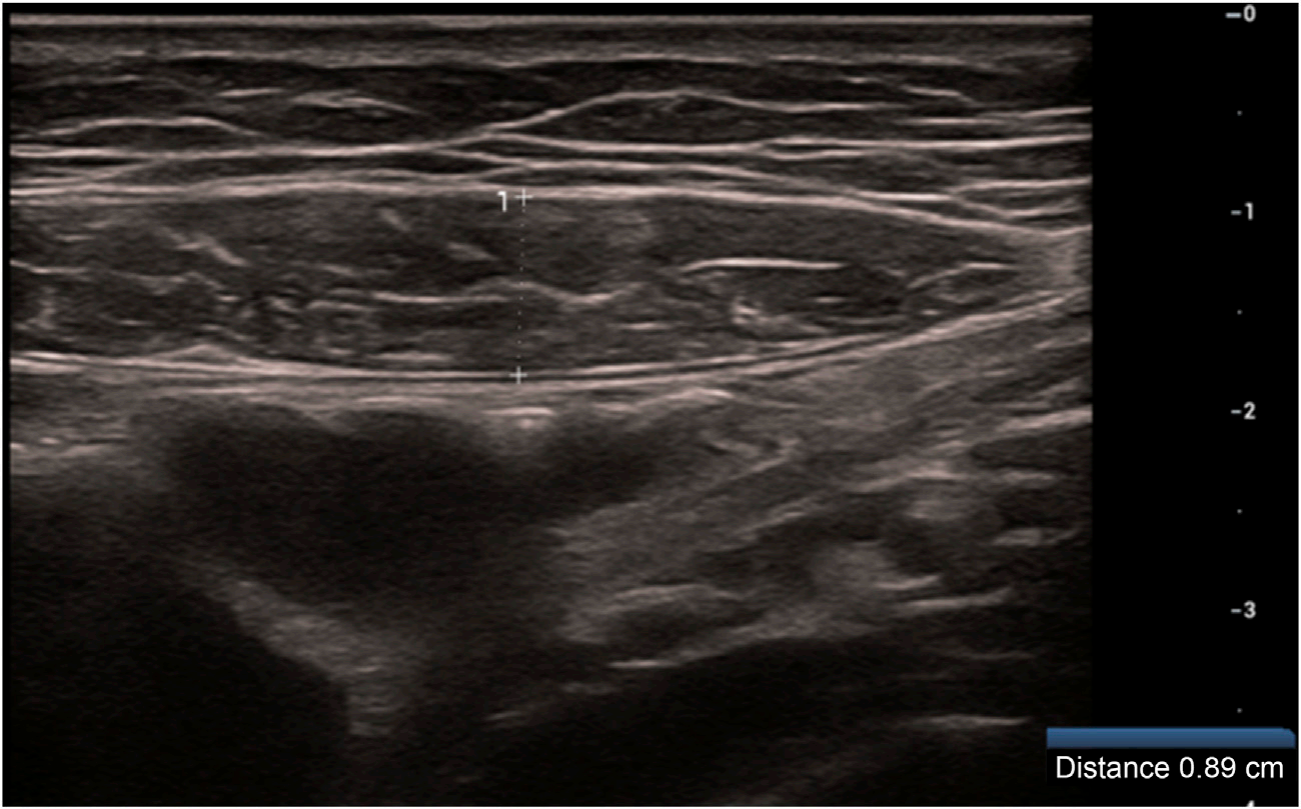
Right rectus abdominis thickness measured at the first trimester (12 weeks).

Gray-scale ultrasonographic measurement of RAT: The right RAT was measured at a transverse section at the level of the umbilicus, and the thickest part of the muscle was selected to measure its thickness. The cursor was placed between the medial margins of the superficial and deep fascia, and the average of three consecutive measurements was obtained (Figure 2).

### SWE examination

**FIGURE 3:**
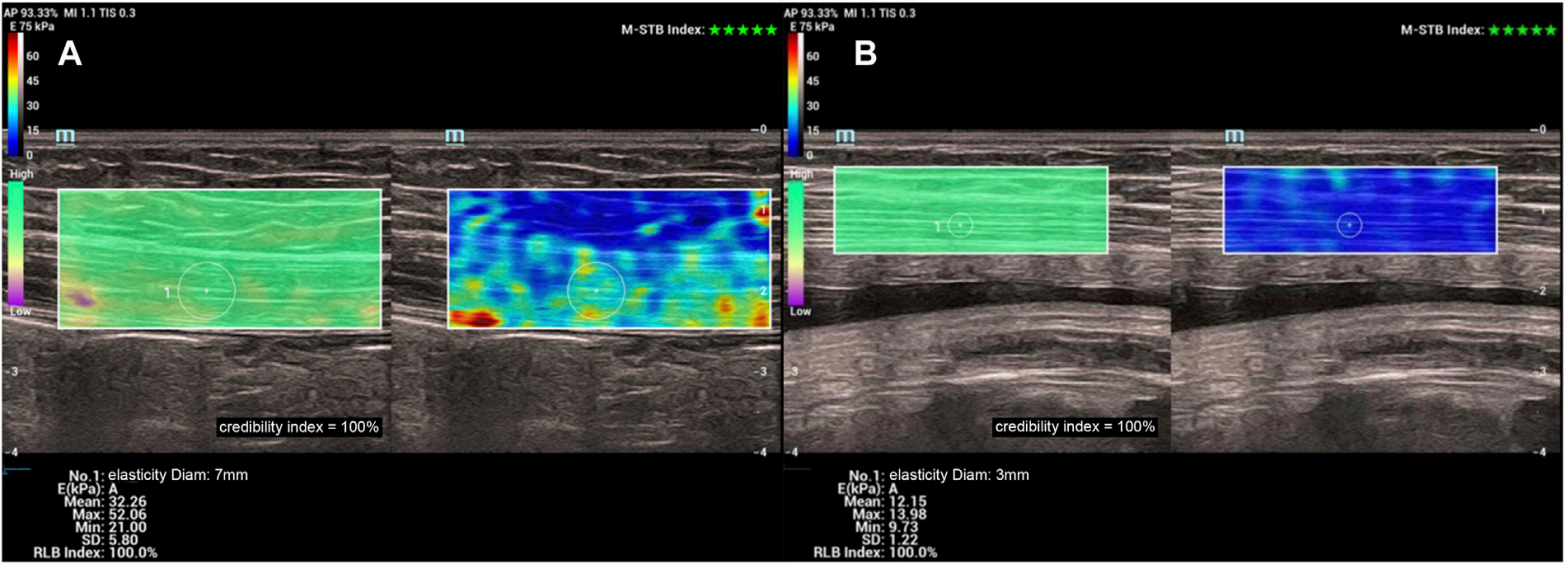
**(A)** Young’s modulus of the right rectus abdominis muscle measured at 12 weeks **(B)** Young’s modulus of the right rectus abdominis muscle measured at 37 weeks.

Measurement of Young’s modulus of rectus abdominis muscle: The Young’s modulus of the right rectus abdominis muscle was uniformly measured by a longitudinal section near the umbilicus level. We ensured that there was an adequate coupling agent between the probe and the skin directly and did not apply pressure to the best possible extent. The SWE sweep mode was turned on and the region of interest (ROI) was selected. The ROI contained the rectus abdominis muscle and a small amount of superficial adipose tissue. The patient was instructed to hold her breath for 3–5 s. When the color of the quality control image on the left side was consistent, the confidence index was >90%, and the upper right corner of the motion stability index was more than four green stars, the elastography image was frozen after it was stable. The Q-BOXTM quantitative measurement tool provided with the machine was used to select the elastography image of the rectus abdominis muscle belly in the ROI range, and the Young’s modulus was measured. The mean Young’s modulus within the measurement range was calculated. Three images were recorded from each volunteer, and the parameter was measured from each of them, resulting in the average of three Q-boxes (Figure 3).

### Collection of other clinical information

The clinical data of pregnant women were collected at the time of documentation and hospitalization for delivery, including age, body height, mass in the first trimester, body mass in the third trimester, fundal height, abdominal circumference, mode of delivery, and newborn’s birth weight.

### Statistical analysis

Statistical analysis was performed using SPSS 26.0 software (IBM Corp., Armonk, United States), and the measurement data are expressed as mean ± standard deviation ( $\bar{x}$ ± s). The differences in IRD, RAT, and Young’s modulus measured at different time points were compared using Friedman’s test. Pairwise comparison was performed using the Bonferroni method (R E, 1990) if differences were present. In multiple comparisons, the Bonferroni method utilizes the t-distribution as the test distribution. However, when utilizing a test level of 0.05 for all comparisons, there is a 5% probability of committing Type I errors in each individual comparison. To address this, the Bonferroni method adjusts the test level by dividing the initial probability of 0.05 by the total number of comparisons. This adjustment ensures that the overall probability of Type I errors occurring in multiple comparisons remains below 0.05. Mann–Whitney U test was used to compare the mean IRD at the first trimester (12 weeks) between multiparae and primiparae. Correlation analysis of RAT and Young’s modulus at different time points was performed using Pearson correlation analysis. All parameters were analyzed using multiple linear regression to determine the risk and protective factors affecting IRD (test level α = 0.05).

## Results

### Basic information

A total of 205 women volunteered to enroll in this study. During follow-up, 2 women terminated their pregnancies, 1 had premature labor, 1 opted for labor induction owing to fetal malformation, 2 were transferred to other hospitals for delivery, and 28 women voluntarily withdrew from the study. Finally, 171 women were included in the study, and their characteristics are listed in Table 1.

**TABLE 1:** Characteristics of 171 pregnant/postpartum study participants.

Clinical variables	N = 171
Age	31 ± 3
Body height	163.56 ± 5.00 cm
Weight at first trimester	57.24 ± 7.98 kg
Weight at delivery	68.73 ± 8.18 kg
BMI at first trimester	21.37 ± 2.61 kg/m^2^
BMI at delivery	25.68 ± 2.72 kg/m^2^
Fundal height	33.90 ± 2.33 cm
Abdominal circumference	98.41 ± 5.29 cm
Primipara	149
Multipara	22
Delivery mode	Natural delivery 122
	Cesarean section 49
Neonate weight	3361.37 ± 382.33 g

BMI, body mass index.

### Variation patterns in the rectus abdominis parameters during pregnancy

**FIGURE 4:**
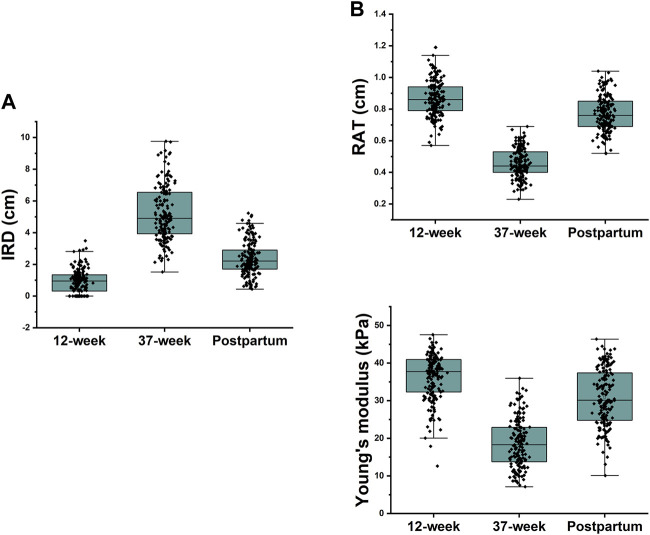
**(A)** 37-week IRD was significantly higher than the first trimester IRD and decreased at 6 weeks postpartum, but it was still higher than the 12-week IRD; **(B)** Rectus abdominis thickness and Young’s modulus were significantly lower at 37 weeks than at 12 weeks and recovered at 6 weeks postpartum but were still lower than those at 12 weeks.

The IRD, RAT, and Young’s modulus of the rectus abdominis showed significant differences among different periods (*p* < 0.001 for all). The IRD at 37 weeks (5.18 ± 1.71 cm) was significantly higher than that at 12 weeks (0.92 ± 0.73 cm), which decreased at 6 weeks postpartum (2.38 ± 1.05 cm); however, it was still higher than that at 12 weeks (*p* < 0.001). RAT and Young’s modulus decreased significantly at 37 weeks (0.46 ± 0.09 cm, 18.68 ± 6.40 kPa) compared with those at 12 weeks (0.87 ± 0.11 cm, 36.31 ± 6.06 kPa) and then recovered at 6 weeks postpartum (0.77 ± 0.11 cm, 30.89 ± 7.52 kPa), but the values were lower than those at 12 weeks (*p* < 0.001) (Table 2). To show the variation trends in the parameters of the rectus abdominis muscle during pregnancy, we plotted box plots (Figure 4).

**TABLE 2:** Comparison of rectus abdominis ultrasonographic parameters in 171 women at their two examination sessions in pregnancy and one postpartum.

Period	IRD (cm)	RAT (cm)	Young’s modulus of rectus abdominis muscle (kPa)
Week 12 of gestation	0.92 ± 0.73^a^	0.87 ± 0.11^d^	36.31 ± 6.06^g^
Week 37 of gestation	5.18 ± 1.71^b^	0.46 ± 0.09^e^	18.68 ± 6.40^h^
6 weeks postpartum	2.38 ± 1.05^c^	0.77 ± 0.11^f^	30.89 ± 7.52^i^
Chi-square value	331.593	303.073	240.082
*P*	<0.001	<0.001	<0.001
*p*-value corrected by Bonferroni (Pairwise comparison)	a Compared with b, *p* < 0.001	d Compared with e, *p* < 0.001	g Compared with h, *p* < 0.001
	a Compared with c, *p* < 0.001	d Compared with f, *p* < 0.001	g Compared with i, *p* < 0.001
	b Compared with c, *p* < 0.001	e Compared with f, *p* < 0.001	h Compared with i, *p* < 0.001

### IRD comparison between multiparae and primiparae at 12 weeks of gestation

Mann–Whitney U revealed that IRD at the first trimester (12 weeks) was significantly higher in multiparae than in primiparae (*p* < 0.001) (Table 3).

**TABLE 3:** Comparison of the IRD between multiparae and primiparae at their 12 weeks of gestation.

IRD of multiparae (*n* = 22) (cm)	IRD of primiparae (*n* = 149) (cm)	Mann-whitney U	
		Z	*p*
1.57 ± 0.74	0.82 ± 0.68	−4.197	<0.001

IRD, inter-recti distance.

### Correlation between RAT and Young’s modulus

A positive correlation was observed between RAT and Young’s modulus of rectus abdominis muscles at 12 and 37 weeks of gestation and 6 weeks postpartum (r = 0.489, *p* < 0.001 (12 weeks); r = 0.454, *p* < 0.001 (37 weeks); r = 0.325, *p* < 0.001 (6 weeks postpartum) (Table 4). Correlation coefficients can be interpreted as follows based on the categorization of the absolute value of correlations: negligible (0.0–0.30), low (0.30–0.50), moderate (0.50–0.70), high (0.70–0.90), and very high (0.90–1.00) (Mukaka, 2012). Hence, a weak positive correlation was observed between RAT and Young’s modulus, regardless of the stage of pregnancy.

**TABLE 4:** Correlation between rectus abdominis thickness and Young’s modulus in 171 women at 12 and 37 weeks of gestation and 6 weeks postpartum.

	Rectus abdominis thickness and Young’s modulus at week 12 of gestation	Rectus abdominis thickness and Young’s modulus at week 37 of gestation	Rectus abdominis thickness and Young’s modulus at 6 weeks postpartum
Pearson correlation coefficient	0.489	0.454	0.325
*p*	<0.001	<0.001	<0.001

### Multiple linear regression analysis

The results of multiple linear regression analysis showed that first trimester IRD (β = 0.294, *p* < 0.001), third trimester IRD (β = 0.429, *p* < 0.001), cesarean section (β = 0.146, *p* = 0.003), and fetal weight (β = 0.246, *p* < 0.001) significantly and positively predicted the 6-week postpartum IRD, and postpartum RAT was a negative predictor (β = −0.163, *p* = 0.016) of IRD at 6 weeks postpartum (Y). We obtained the multiple linear regression equation as follows: $Y=-0.878+0.420\times X_{1}+0.263\times X_{2}-1.524\times X_{3}+0.338\times X_{4}+0.001\times X_{5}$ . Analysis of variance result: f = 24.856, *p* < 0.001, the regression model was successfully established with an adjusted R^2^ of 0.716, indicating that the model explained 71.6% of the variation in IRD at 6 weeks postpartum (Table 5).

**TABLE 5:** Multiple linear regression analysis of factors affecting IRD at 6 weeks postpartum.

	B	β	t	*p*	F	Adjusted R^2^
First trimester IRD (X_1_)	0.420	0.294	5.103	<0.001	24.856	0.716
Third trimester IRD (X_2_)	0.263	0.429	7.100	<0.001		
Postpartum rectus abdominis thickness (X_3_)	−1.524	−0.163	−2.437	0.016		
Cesarean section (X_4_)	0.338	0.146	3.072	0.003		
Fetal weight (X_5_)	0.001	0.246	3.758	<0.001		

IRD, inter-recti distance.

## Discussion

In this prospective study, we observed and analyzed variation patterns in rectus abdominis parameters at three time points, starting with the first trimester and following up to 6 weeks postpartum. When the intra-abdominal pressure and estrogen levels decrease after delivery, the thickness and stiffness of the rectus abdominis muscle begins to recover gradually. The results of this study showed that RAT and Young’s modulus of the rectus abdominis muscle at 6 weeks postpartum were greater than those in 37 weeks, but were lower than those in the first trimester (12 weeks). We hypothesized muscle damage to a certain extent owing to the decrease in its RAT and stiffness during pregnancy and the simultaneous decrease in muscle strength and other attributes. The gradual recovery of the RAT and stiffness of the rectus abdominis muscle after delivery was beneficial for the restoration of the muscle status; however, it was lower than that at 12 weeks. This finding indicated that the damage to the rectus abdominis muscle caused by pregnancy is not fully restored to pre-pregnancy levels at 6 weeks postpartum.

There has been controversy regarding the diagnostic criteria for DRA, especially in terms of the diagnostic criteria for postpartum women. In the study conducted by Mota et al. (Mota et al., 2018), the width of the IRD of the linea alba was assessed in primiparous women at four different time points: late pregnancy and three times in the postpartum period. Ultrasound images of 84 primiparous women were used, focusing on three locations along the linea alba (2 cm below the umbilicus and 2 and 5 cm above the umbilicus). Measurements were taken during gestational weeks 35–41 and 6–8, 12–14, and 24–26 weeks postpartum. The normal width of the linea alba was defined using the 20th and 80th percentiles. The normal IRD ranged 54–86 mm (2 cm above the umbilicus, gestational week 35–41) and 17–28 mm (2 cm above the umbilicus, postpartum week 6–8). We compiled the IRD of 171 patients at gestational week 37 and postpartum week 6 and obtained normal ranges of 37 ± 67 mm (above the umbilicus, gestational week 37) and 16–32 mm (above the umbilicus, postpartum week 6). The normal IRD ranges obtained in the two studies are relatively consistent, and any differences observed may be attributed to differences in location of measurement site, length of follow-up time, and sample size. Mona et al. reported that in primiparous women, the IRD can be considered “normal” even with wider values compared to nulliparous women, which is consistent with this study.

Variation patterns in ultrasonographic parameters of the rectus abdominis muscle at each time point showed that the Young’s modulus at 37 weeks was significantly lower than that at 12 weeks. This is the first study to report that the stiffness of the rectus abdominis muscle decreases with gestational age in an observational trial. Changes in hormone levels are also responsible for the formation of DRA. Khowailed et al. (Khowailed et al., 2022) analyzed the effect of the menstrual cycle on the stiffness of medial gastrocnemius and tibialis anterior muscles using SWE. They found that the stiffness of the skeletal muscles during ovulation was lower than that in the follicular phase and that the level of estrogen during ovulation was approximately 10 times higher than that in the follicular phase. In addition, they observed that the level of estrogen during the pregnancy period was thousands of times higher than that during non-pregnancy. Therefore, the stiffness of the rectus abdominis muscle also decreases under the influence of high levels of estrogen. This change has some physiological significance because the rectus abdominis muscle becomes softer during pregnancy, making it easier for stretching, which helps to enlarge the abdominal cavity to accommodate the growing fetus.

Typically, the areas within the human body where shear waves propagate as guided waves include the arterial walls, bladder walls, corneas, and Achilles tendon (Guo-Yang et al., 2018). The Achilles tendon, in particular, exhibits a longer shear wavelength due to its high stiffness (approximately 400–1300 kPa), which surpasses the thickness of the propagation medium. Consequently, when researching these areas, the impact of guided waves must be taken into consideration (Brum et al., 2014). On the other hand, the stiffness of the rectus abdominis muscle is significantly lower than that of the Achilles tendon. In this study, the rectus abdominis muscle’s stiffness was estimated to be around 20 kPa, and the calculated shear wavelength was approximately 3 mm, which fell within the thickness of the rectus abdominis muscle. Hence, in theory, the influence on the measured Young’s modulus in this study could be disregarded.

We used multiple linear regression analysis to show that first- and third trimester IRD, cesarean section, and fetal weight were risk factors for early postpartum DRA, whereas postpartum RAT was a protective factor. Cesarean delivery and fetal weight have been previously reported as risk factors for postpartum DRA (Turan et al., 2011). However, our study found that first- and third trimester IRD at the same time are risk factors for postpartum DRA. This finding is an innovative aspect of this study, and the IRDs at these two time points have their own special clinical values and are important for predicting postpartum DRA. For example, when a woman in the third trimester has an IRD that is too large, she is advised to wear a maternity belt and take necessary interventional measures in the early postnatal period.

In the first trimester (12 weeks), the fetus is small and the uterus is still in the pelvis, which does not cause compression or damage to the rectus abdominis muscle at the level of the umbilicus. During this time, IRD can be used to approximate the pre-pregnancy state of the rectus abdominis muscle. The incidence of postpartum DRA has been reported to be higher in multiparae than in primiparae (Wu et al., 2021), probably because the DRA status of multiparae is more severe than that of primiparae in the first trimester. The results of our study also showed that the IRD of multiparae was significantly higher than that of primiparae in the first trimester (12 weeks). Therefore, the effect of pregnancy on the rectus abdominis muscle in women can be reflected by the IRD in the first trimester, with larger values representing a poorer initial state of the muscle.

Pregnancy is considered as full term at 37 weeks. At this point, the weight of the fetus is almost similar to that at birth. Therefore, the state of the rectus abdominis muscle at 37 weeks is similar to that before delivery. During pregnancy, as the fetus grows, intra-abdominal pressure increases and the DRA status gradually worsens. Several factors, such as maternal weight, fetal weight, fundal height, and increase in abdominal circumference during pregnancy, affect the degree of aggravation of DRA in the third trimester, and increase the difficulty of the analysis. Therefore, we measured the easiest and most intuitive IRD in the third trimester. The reason is that regardless of the factors during pregnancy, they will be finally reflected as changes in IRD and third trimester IRD can reflect the degree of exacerbation of DRA during pregnancy.

Furthermore, our study found postpartum RAT to be a protective factor against DRA in the early postpartum period. Kelly et al. (Kelly et al., 2022) observed a strong correlation between RAT and its transverse area in both women (r = 0.758) and men (r = 0.715), suggesting that muscle thickness is a valid indicator of muscle size. Peter et al. (Soldos et al., 2021) used SWE to analyze the relationship among thickness, stiffness, and torque of the vastus lateralis muscle in athletes and non-athletes. During isometric maximal contraction of the muscle, its thickness and stiffness were significantly greater in athletes than in non-athletes. Moreover, force measurement data showed that the muscle torque of athletes during maximal isometric contraction was significantly larger than that of non-athletes, and there was a positive correlation among thickness, stiffness, and torque of the muscles. We analyzed the correlation between RAT and Young’s modulus at three time points and found that these two parameters exhibited a weak positive correlation, which is consistent with the results of the study by Peter et al. Therefore, we believe that the RAT and stiffness of the rectus abdominis muscle may reflect physiological characteristics such as rectus abdominis muscle strength. Thicker muscles have relatively higher stiffness and muscle strength (Misirlioglu et al., 2021; Yoshimura et al., 2022), which beneficial for counteracting the traction of the growing abdominal cavity on both rectus abdominis muscles during pregnancy and facilitates the recovery of the muscle and shrinkage of the IRD during postpartum.

Guo et al. investigated the application of two-dimensional ultrasound and SWE in assessing DRA at various gestational periods (Guo et al., 2024). Their study showed that the Young’s modulus of the rectus abdominis was positively correlated with the thickness of rectus abdominis (r = 0.408), consistent with the results of our study. They also found that the Young’s modulus of the rectus abdominis was negatively correlated with the IRD (r = 0.515). He et al. (He et al., 2021) demonstrated that the shear wave speed of rectus abdominis showed a positive correlation with IRD (0.574), a finding contradictory to the results obtained by Guo et al. This study did not find a correlation between rectus abdominis stiffness and IRD, and the following reasons were stated for this. First, we did not use the same instrumentation; therefore, there may be differences in the methods used to measure the Young’s modulus of rectus abdominis or shear wave speed. Second, the studies differed in terms of the sample selection method and sample size. Guo et al. and He et al. conducted cross-sectional studies in which a subset of women was selected depending on the corresponding gestational week or postpartum period. In this study, we continuously observed and followed-up the patients from the first trimester of pregnancy up to the postpartum period. Furthermore, He et al. analyzed only 36 patients with DRA, and Guo et al. did not specify whether correlation analysis was conducted on samples in a specific period or on the overall sample.

As researchers continue to pay more attention to DRA, there are increasing numbers of studies on the treatment modalities for this condition (Michalska et al., 2018; Kaya and Menek, 2023; Redi et al., 2023). However, only IRD has been used for efficacy assessment, and assessment of the biological parameters of the rectus abdominis muscle is lacking. If dynamic examination of RAT and stiffness can be included in the evaluation of DRA treatment, the variation patterns before and after treatment can be more comprehensively monitored. This examination will allow a more objective assessment of which treatment has better efficacy for DRA.

There are some limitations of this study. This is a single-center study with a short postpartum follow-up period, and we only assessed the status of the rectus abdominis muscle at 6 weeks postpartum. At that time, the rectus abdominis muscles of some mothers had not recovered to their optimal state. Moreover, because our study was conducted in pregnant women, we did not assess the consistency and reliability of the measurements for two-dimensional ultrasonography and elastography owing to ethical considerations. In addition, we measured only the thickness and Young’s modulus of the right rectus abdominis muscle and IRD at one site in the superior umbilical border. Furthermore, we excluded pregnant women with multiple pregnancies as some studies have shown that multiple pregnancies are a risk factor for DRA (Turan et al., 2011). Moreover, the gestational age at delivery varies widely among pregnant women with multiple pregnancies, and a significant proportion of those with multiple pregnancies deliver before 37 weeks. Therefore, we excluded women with multiple pregnancies from this study.

## Conclusion

The risk and protective factors of DRA discovered through this study may guide pregnant women’s protection and treatment during the perinatal period. Our study identified a variation pattern in the thickness and stiffness of the rectus abdominis muscle during pregnancy and postpartum, which provides a new direction for further examination of the mechanism of DRA formation. However, owing to the limitations of this study, more research is needed to validate the findings, and it is especially important to follow-up patients in the late postpartum period.

## Data Availability

The raw data supporting the conclusion of this article will be made available by the authors, without undue reservation.
